# Bonding of Neuropeptide Y on Graphene Oxide for Drug Delivery Applications to the Central Nervous System

**DOI:** 10.1021/acsanm.2c03409

**Published:** 2022-12-02

**Authors:** Giada Cellot, Lucas Jacquemin, Giacomo Reina, Audrey Franceschi Biagioni, Mario Fontanini, Olivier Chaloin, Yuta Nishina, Alberto Bianco, Laura Ballerini

**Affiliations:** † International School for Advanced Studies, SISSA, Via Bonomea n. 265, 34136Trieste, Italy; ‡ CNRS, Immunology, Immunopathology and Therapeutic Chemistry, UPR 3572, University of Strasbourg ISIS, 67000Strasbourg, France; § Graduate School of Natural Science and Technology and Research Core for Interdisciplinary Sciences, Okayama University, Tsushimanaka, Kita-ku, Okayama700-8530, Japan

**Keywords:** carbon nanomaterials, 2D materials, functionalization, drug delivery, brain diseases

## Abstract

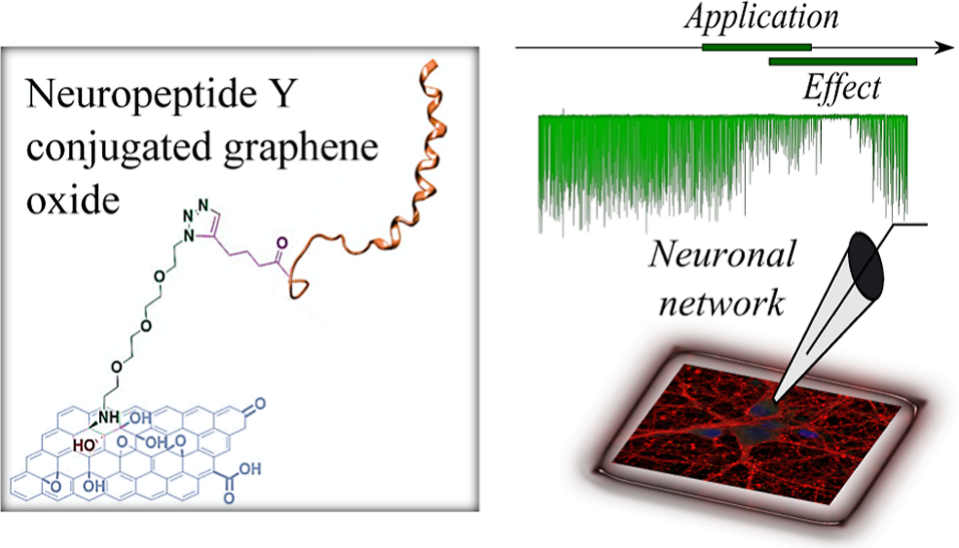

Nanoscale graphene-based materials (GBMs) enable targeting subcellular structures of the nervous system, a feature crucial for the successful engineering of alternative nanocarriers to deliver drugs and to treat neurodisorders. Among GBMs, graphene oxide (GO) nanoflakes, showing good dispersibility in water solution and being rich of functionalizable oxygen groups, are ideal core structures for carrying biological active molecules to the brain, such as the neuropeptide Y (NPY). In addition, when unconjugated, these nanomaterials have been reported to modulate neuronal function *per se*. Although some GBM-based nanocarriers have been tested both in vitro and in vivo, a thorough characterization of covalent binding impact on the biological properties of the carried molecule and/or of the nanomaterial is still missing. Here, a copper(I)-catalyzed alkyne–azide cycloaddition strategy was employed to synthesize the GO–NPY complex. By investigating through electrophysiology the impact of these conjugates on the activity of hippocampal neurons, we show that the covalent modification of the nanomaterial, while making GO an inert platform for the vectorized delivery, enhances the duration of NPY pharmacological activity. These findings support the future use of GO for the development of smart platforms for nervous system drug delivery.

## Introduction

During the last decades, thanks to their mechanical, electrochemical, and optical properties^1−3^ graphene-based materials (GBMs) have been increasingly engineered for biomedical applications,^4^ with a particular focus on the neuroscience area of intervention.^5,6^ The nanoscale dimension of these materials, matching that of the nervous tissue subcellular elements such as synaptic vesicles or dendritic spines,^7−9^ has boosted their development as highly selective nanocarriers specifically targeting basic brain functional units instrumental to neuronal operativity. Such a characteristic combined with GBM large surface area, which favors the anchoring of chemical compounds,^10^ might be useful for the development of GBM-based nanocarrier systems for targeted drug delivery in the central nervous system (CNS).

Among GBMs, GO has received extensive attention in this field due to its good dispersibility in aqueous media,^11−13^ a feature crucial for drug delivery applications, and to its wealth of oxygen containing functional groups making it easy to bind biomolecules such as genes, drugs, antibodies, or peptides.^14^ In addition, pristine GO nanoflakes have been reported to target precisely synapses tuning neuronal activity in vitro and in vivo^15−19^ making this nanomaterial interesting not only for the delivery of drugs but also as the active ingredient *per se*.

Although both in vitro and in vivo studies reported that GO conjugated with therapeutic molecules can be used as drug nanocarriers (reviewed in ref (20)), it remains to be elucidated whether the interactions between GO and the carried bioactive molecule (e.g., a drug) composing the delivery system affect either the drug specificity or the impact of GO on neuronal function. In this context, it is unclear (i) whether GO retains the biological activity on synapses observed for the unconjugated nanomaterial after being covalently conjugated with a bioactive molecule, (ii) if the therapeutic efficacy of the carried molecule is changed by the binding to GO, and (iii) if the presence of the nanocarrier alters the pharmacokinetic properties of the pharmacologically active molecule of the conjugates. In this work, we address all these questions by covalently binding an active peptide, the human neuropeptide Y (NPY), a modulator of neuronal transmission^21−23^ involved in several physiological CNS functions,^24^ to GO and investigating the ability of GO–NPY to modify synaptic activity when delivered to neuronal networks in dissociated hippocampal cultures. By using acute, subacute, and chronic applications of the conjugate to dissect the biological effects due to the nanomaterial from those due to the neuroactive peptide, our electrophysiological experiments showed that GO, once chemically modified, loses the capacity to modulate neuronal function when compared to pristine GO. Differently, NPY, once bound to GO, not only retains its biological activity but also presents a prolonged effect respect to free NPY, suggesting an enhanced pharmacokinetic profile. These findings might prompt the use of GO as platform for the development of nanomaterial-based drug delivery systems for the treatment of neurodisorders.

## Experimental Section

### Materials

Amino acids and resin were purchased from Iris Biotech. 11-Azido-3,6,9-trioxaundecan-1-amine (TEG-N_3_) was purchased from Sigma-Aldrich, copper(II) sulfate pentahydrate from Carlo Erba, sodium l-ascorbate from Acros, and 5-hexynoic acid from Alfa Aesar. The solvents were obtained from commercial suppliers and used without purification. Water was purified using a Millipore filter system Milli-Q and free endotoxin Polisseur Biopak. For dialysis, MWCO 12,000–14,000 Da membranes were purchased from Spectrum Laboratories, Inc. All chemicals for biological experiments were purchased from Sigma-Aldrich if not differently stated.

### Methods

Attenuated total reflection–Fourier transform infrared (ATR–FTIR) was performed using a Thermo Scientific FTIR spectrometer equipped with an ATR accessory (diamond ATR polarization accessory with 1 reflection top-plate and pressure arm) and coupled to the software Nicolet iS 50. The pressure arm was used for all solid samples. The number of scans was set at 64. Samples were loaded on the reflection top-plate at a quantity sufficient to cover the entire diamond surface. X-ray photoelectron spectroscopy (XPS) analysis was performed on a Thermo Scientific K-Alpha XPS with a basic chamber pressure of 10^–8^ to 10^–9^ bar and an Al anode as the X-ray source (1486 eV). The samples were analyzed as powder pressed onto a scotch tape (3MTM EMI Copper Foil Shielding Tape 118). Spot size of 400 μm was used for analysis. The survey spectra are an average of 10 scans with a pass energy of 200.00 eV and a step size of 1 eV. For each sample, the analysis was repeated three times. A flood gun was turned on during analysis. Thermogravimetric analysis (TGA) was performed on a TGA1 (Mettler Toledo) apparatus from 30 to 900 °C with a ramp of 10 °C/min under N_2_ using a flow rate of 50 mL/min and platinum pans. For GO materials, samples were lyophilized before analysis. Circular dichroism (CD) spectra were recorded with a J-810 Jasco spectropolarimeter. Each spectrum was recorded at 1 nm resolution after 16 accumulations from 192 to 260 nm. For this set of experiments, GO nanomaterials were diluted in a mixture of trifluoroethanol (TFE) and phosphate buffer solution (PBS) in a ratio 1:1 to obtain a final concentration at 0.05 mg/mL. 0.5 mL of solution was directly transferred into a 0.2 mm path length quartz cuvette. The spectra were obtained by subtraction of the appropriate blank. Peptide helical fraction was calculated based on this equation
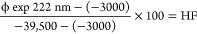

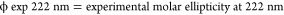


### Synthesis of NPY Peptide

Human NPY peptide (YPSKPDNPGEDAPAEDMARYYSALRHYINLITRQRY-NH_2_) terminated at the N-terminus with 5-hexynoic acid was prepared using Fmoc chemistry protocols on a multichannel peptide synthesizer.^25^ Sidechain deprotection and cleavage of the peptides from the solid support were performed by treatment with reagent K (88% TFA v/v, 2% triisopropylsilane v/v, 5% dithiothreitol w/v, and 5% water v/v) for 150 min at 20 °C. The peptide was purified by reversed-phase HPLC (RP-HPLC) using a preparative HPLC system (Waters) on a Nucleosil C18 (1 × 30 cm) column (Macherey Nagel). The elution was achieved with a linear gradient of aqueous 0.1% TFA (A) and 0.08% TFA in acetonitrile (B) at a flow rate of 6 mL/min with UV detection at 230 nm. The purity of the peptide was controlled by analytical RP-HPLC on a Waters instrument (Waters Alliance) with a Nucleosil C18 5 μm column (150 × 4.6 mm) using a linear gradient of 0.1% TFA in water and acetonitrile containing 0.08% TFA at a flow rate of 1.2 mL/min. The integrity of the peptide was assessed by LC/MS using a Thermo Finigan LCQ.

### Synthesis of GO–NPY

#### Synthesis of GOTEG-N_3_

For the functionalization with TEG-N_3_, GO were dispersed in endotoxin-free Milli-Q water at 1.43 mg/mL. In order to reduce the agglomeration of the flakes during the functionalization process, the dispersion of GO (7 mL) was stirred using ULTRA-TURRAX T 10 at low speed (power 3; ref (26)). Subsequently, TEG-N_3_ (11 mg) in endotoxin free Milli-Q water (3 mL) have been added dropwise using a pipette to reach a final concentration of GO at 1 mg/mL. After the ULTRA-TURRAX has been switched off, the reaction was further stirred for 24 h via magnetic agitation. Subsequently, the unreacted TEG-N_3_ has been removed via dialysis using endotoxin free Milli-Q water (3 days). The loading of TEG-N_3_ was established from the data obtained by XPS.

#### Coupling with NPY Peptide

A copper-catalyzed azide–alkyne click cycloaddition (CuAAC) was carried out on graphene oxide by a modified protocol from the literature.^27^ The reaction was performed in pure water. A solution of modified NPY (3 mg), sodium l-ascorbate (0.386 mg), and copper(II) sulfate pentahydrate (0.40 mg) was prepared in 1 mL of endotoxin free Milli-Q. Subsequently, this solution was added dropwise using a pipette to a magnetically stirred solution of GOTEG-N_3_ (3 mL at 3 mg/mL) dispersed in endotoxin free Milli-Q. The reaction was stirred for 4 days. The unreacted reagents were removed via dialysis using endotoxin free Milli-Q water (3 days, 3 transfer to fresh water per day). The loading of peptide was measured from the data obtained by XPS.

### Preparation of Rat Dissociated Hippocampal Cultures

Dissociated hippocampal cultures were prepared from 2 to 3 days postnatal (P2–P3) rats.^28^ All procedures were done in agreement with the Italian law (decree 26/14) and the EU guidelines (2007/526/CE and 2010/63/UE). The animal use was authorized by the Italian Ministry of Health (authorization number: 22DAB.NYQA) and approved by the local veterinary authorities. After hippocampus isolation, cells were enzymatically and mechanically dissociated and then seeded on poly-l-ornithine-coated glass coverslips (24 × 12 mm^2^, Kindler, EU) at a density of 150,000 cells/mL. Neuronal cultures were maintained in stable conditions (37 °C, 5% CO_2_) in a medium consisting of MEM (Gibco), 35 mM glucose, 1 mM apo-transferrin, 15 mM HEPES, 1 mM insulin, 4 μM biotin, 3 μM vitamin B12, 500 nM gentamicin, and 10% fetal bovine serum (FBS; Invitrogen). After 2 days, the culture medium was replaced with one containing 1B-arabinofuranosilcitosina (Ara-C, 5 μM), to prevent glial over-proliferation, and then changed every 3 days.

### Immunofluorescence Labeling of Dissociated Hippocampal Cultures

Immunofluorescence staining was performed as described previously.^29^ Hippocampal cells were fixed in PBS containing 4% PFA for 20 min at RT. Cells were permeabilized with 1% Triton X-100 for 30 min, blocked with 5% FBS in PBS for 30 min at room temperature (RT), and incubated with primary antibodies for 60 min. The primary antibodies used were mouse polyclonal anti-β-tubulin III (Sigma, 1:250 dilution), rabbit polyclonal anti-NPY1R (Abcam Plc, 1:500 dilution), and rabbit polyclonal anti-NPY2R (Thermo Fisher, dilution 1:2000). After the primary incubation and PBS washes, neurons were incubated for 60 min with the secondary antibodies Alexa Fluor 488 goat anti-rabbit (Invitrogen, dilution 1:500), Alexa Fluor 594 goat anti-mouse (Invitrogen, dilution 1:500), Alexa Fluor 488 goat anti-guinea pig (Invitrogen, dilution 1:500), and DAPI (Invitrogen, dilution 1:200) to stain the nuclei. Samples were mounted in VECTASHIELD (Vector Laboratories) on 1 mm thick coverslips. Image acquisition was performed using a confocal microscope (Leica Microsystems GmbH, Wetzlar, Germany) with 63× (1.4 NA) magnification (*Z*-stacks were acquired every 150 nm).

### Electrophysiological Recordings

Neuronal activity was recorded from dissociated hippocampal cultures after 10–15 days of differentiation in vitro through single cell patch clamp technique. Voltage clamp whole-cell recordings were performed at RT with pipettes (5–7 MΩ) containing (in mM): 120 K gluconate, 20 KCl, 10 HEPES, 10 EGTA, 2 MgCl_2_, 2 Na_2_ATP, pH 7.3. The extracellular solution contained (in mM) the following: 150 NaCl, 4 KCl, 2 CaCl_2_, 1 MgCl_2_, 10 HEPES, 10 glucose, pH 7.4. Cultures were mounted on a chamber and visualized with an inverted microscope (Eclipse TE-200, Nikon, Japan). During experiments, neurons were continuously perfused with extracellular solution through a gravity-based perfusion system at a rate of 1 mL/min. Recordings were performed through a Multiclamp 700B patch amplifier (Axon CNS, Molecular Devices) with a sampling rate of 10 kHz. Data were acquired using pClamp 10.2 software (Molecular Devices LLC, San Jose, CA, USA). The stability of the recording was checked by repetitively monitoring the series resistance (<20 MΩ and not compensated) during the experiments and cells showing 15% changes were excluded. Input resistance and cell capacitance were measured online with the membrane test feature of the pClamp software. These passive membrane properties, indicators of neuronal health,^18^ were not changed between different groups of treatments. In acute puff applications experiments, capacitance was 101 ± 8 pF in control, 81 ± 8 pF in GO-treated, 88 ± 11 pF in GO–NPY-treated, and 87 ± 12 pF in NPY-treated samples, while input resistance was 347 ± 53 MΩ in control, 469 ± 120 MΩ in GO-treated, 529 ± 75 MΩ in GO–NPY-treated, and 368 ± 50 MΩ in NPY-treated samples.

For sub-acute applications, capacitance was 98 ± 34 pF in control, 104 ± 32 pF in GO–NPY-treated, and 94 ± 21 pF in NPY-treated samples, while input resistance was 453 ± 263 MΩ in control, 387 ± 274 MΩ in GO–NPY-treated, and 468 ± 211 MΩ in NPY-treated samples.

For chronic applications, capacitance was 116 ± 8 pF in control, 94 ± 14 pF during GO–NPY treatments, 100 ± 17 pF during NPY treatments, 110 ± 9 pF in GO–NPY washed out, and 99 ± 11 pF in NPY washed out samples. Input resistance was 316 ± 158 MΩ in control, 398 ± 83 MΩ during GO–NPY treatments, 498 ± 101 MΩ during NPY treatments, 337 ± 66 MΩ in GO–NPY washed out, and 417 ± 80 MΩ in NPY washed out samples.

Recordings of spontaneous postsynaptic activity were performed at a holding potential of −56 mV (not corrected for liquid junction potential, which was −14 mV). The recorded traces were analyzed offline with the AxoGraph 1.4.4 event detection software (Axon CNS, Molecular Devices) to analyze spontaneous postsynaptic currents (sPSC) frequency and amplitude.

### Live Calcium Imaging with GCaMP7f

pGP-AAV-*syn*-jGCaMP7f-WPRE was a gift from D. Kim and GENIE Project (Addgene plasmid no. 104488; http://n2t.net/addgene:104488; RRID:Addgene_104488). AAV1/2 viruses were prepared as described previously.^30^ For infection, dissociated hippocampal neurons were incubated with virus diluted 1:5000 in a culture medium at days in vitro (DIV) 9 and used for calcium imaging experiments at 13 DIV.

Neurons expressing GCaMP7f were recorded in a custom 3D-printed perfusion chamber mounted on a Nikon microscope (Nikon Eclipse Ti2 microscope endowed with a Nikon Intensilight Hg lamp and an Andor Zyla sCMOS) in extracellular recording solution (as that used for electrophysiology). GCaMP7f fluorescence was visible with a 20× S Plan Fluor ELWD NA 0.45 objective with 5 ms of exposure, when using ND filter 32 for the excitation light and imaged with the latter objective at a sampling rate of 6.6 fps and 4 × 4 binning.

The acquired time series of images recorded from the selected field (size, 636.4 μm by 636.4 μm) were analyzed in Fiji choosing 20 to 30 ROIs of ∼ Ø15 to 20 μm placed onto the cell soma, and the obtained fluorescence traces were processed in Clampfit 10.7 (Molecular Probes).

### Acute, Sub-Acute, and Chronic Applications of Conjugate

The conjugate and the related controls were applied through different types of treatments: acute, sub-acute, and chronic ones. In acute applications, by using a Picospritzer (PDES-02DX; NPI electronic GmbH, Germany), an injection of pressurized air (500 ms, 0.5 psi) was used to deliver a puff of solution containing the nanomaterials or NPY to hippocampal neurons. The puff pipette, located at a distance of 200 μm from the recorded cell, was filled with extracellular solution (control), GO (200 μg/mL), GO–NPY (200 μg/mL and 10 μM, respectively), or NPY (10 μM), all diluted in extracellular saline solution. Considering the volume (1 mL) of the extracellular solutions in the recording chamber, the final concentration of conjugates (or control solutions) reaching the patch-clamped neurons was 10% of that present in the puff pipette.^29^ PSCs were recorded before and after (10 min each) the local ejection.

Regarding sub-acute treatments, after monitoring baseline neuronal activity for 10 min conjugates (20 μg/mL and 1 μM, for GO and NPY, respectively) or NPY (1 μM) were applied through the perfusion system for 5 min and then washed out for additional 5 min.

For chronic applications, neurons were incubated with the conjugates or NPY (same concentrations as in sub-acute treatments) in the extracellular solution for 30 min prior to recording. The duration of conjugate or NPY effects was measured upon incubation of cells and subsequent perfusion with fresh extracellular solution for 10 min prior to recording.

### Statistical Analysis

All values from samples undergone to the same experimental treatment were pooled together and expressed as mean ± SEM with *n* = number of neurons, if not otherwise indicated. D′Agostino & Pearson omnibus normality test was applied to evaluate the statistical distribution of the data sets. In the case of data distributed as in a Gaussian distribution we used one-way analysis of variance followed by Tukey’s multiple comparison tests, otherwise we used Kruskal–Wallis test and Dunn’s multiple comparisons test. Statistical significance was considered for *P* < 0.05.

## Results

**Scheme 1:**
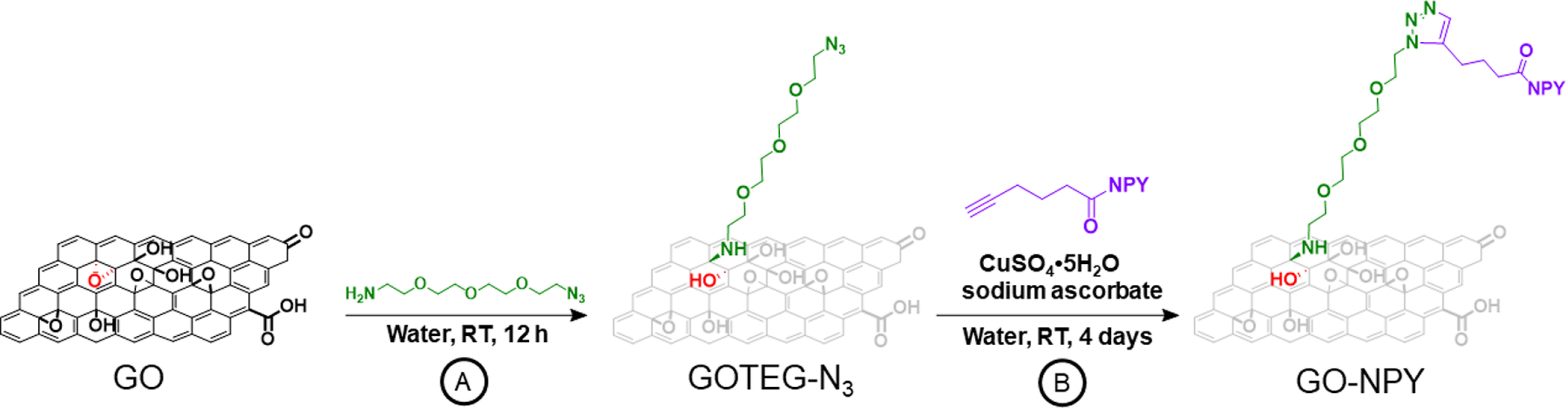
Functionalization of GO by Epoxide Ring Opening (A), Followed by the Copper Catalyzed Click Reaction (B)

Based on our previous results^15,17−19,29^ describing the impact of graphene materials on neuronal functions, we have selected a nanoscale GO obtained by Hummers’ oxidation of graphite for combination with the NPY peptide. The single layer of GO sheets possessed a size distribution centered at 430 nm.^31^ For the covalent functionalization, we first prepared GOTEG-N_3_ via epoxide ring opening (Scheme 1). In particular, TEG-N_3_ was chosen to introduce a spacer with a water soluble and flexible chain between the graphitic surface and the peptide (Scheme 1A). GOTEG-N_3_ was prepared following a method previously reported by our team based on the “ultramixing” technique.^26,32^ “Ultramixing” helps to disperse the material via a high circumferential speed, avoiding the aggregation, induced by charge destabilization between the negatively charge surface of GO and the positive primary amine on the functional PEG chain. NPY peptide was modified at the N terminus with a short alkyne chain, 5-hexynoic acid.

The triple bond carried by NPY allowed a selective chemical ligation to GOTEG-N_3_ catalyzed by copper sulfate and sodium ascorbate (Scheme 1B). We have designed this synthetic strategy because it is highly selective, efficient, and allowed to work under mild conditions.

**Figure 1:**
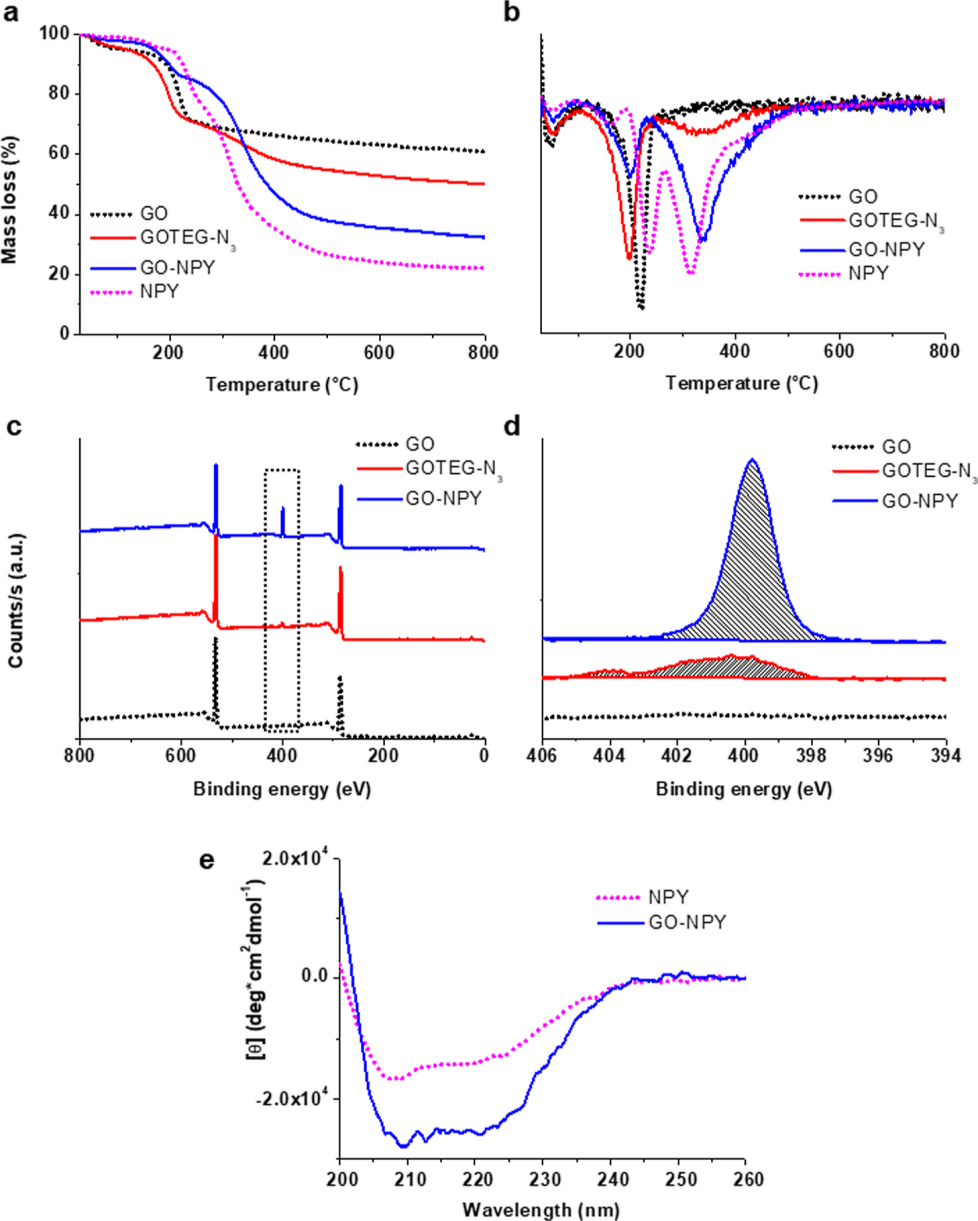
Characterization of the conjugate. (a) TGA of GO, GOTEG-N_3_, GO–NPY, and NPY, (b) derivative thermogravimetry of GO, GOTEG-N_3_, GO–NPY, and NPY, (c) XPS survey spectra of GO, GOTEG-N_3_, and GO–NPY, and (d) XPS high-resolution spectra N 1s of GO, GOTEG-N_3_, and GO–NPY. (e) CD of NPY and GO–NPY in 1:1 PBS/TFE. In the case of GO–NPY, GOTEG-N_3_ was used as blanc.

The resulting dispersion was purified by dialysis, achieving the final GO–NPY conjugate. GO–NPY, GO, and the intermediate GOTEG-N_3_ were characterized by TGA, XPS (Figure 1), and FTIR (Figure S1). SEM of GO (Figure S2) allowed us to visualize the single layers and to calculate the size distribution, as already reported in a previous paper where we used the same starting material.^31^ TGA was used to compare the weight loss after each synthetic step. The thermal profile was analyzed by derivative thermogravimetry (DTG; Figure 1a,b). The thermal profile of GO in an inert atmosphere (black line) shows the typical two-step degradation profile, with a first small step at around 100 °C and a second one at about 200 °C associated to loss of water and labile oxygenated functions,^33^ respectively. GOTEG-N_3_ curve (red line) displays two similar peaks, with the degradation profile at 200 °C slightly shifted below this temperature. This shift can be explained by the conversion of the epoxide groups into secondary amines less thermally stable.^33^ TGA profile of NPY (pink line) displays a mass loss at around 300 °C clearly visible also in the DTG analysis. Similarly for GO–NPY, the mass loss around 330 °C (broad peak in DTG) is observed corresponding to the degradation of the peptide onto the nanomaterial surface. The increase of the degradation temperature of NPY can be ascribed to the covalent grafting of the peptide onto the GO surface that enhances the thermal stability as already observed with other polymers.^34^ Subsequently, we used XPS to study the modification of the surface chemistry of the different materials after each grafting step. XPS survey (Figure 1c) reveals that GO is mainly composed by carbon and oxygen, with a N content of only 0.5% (Table S1). The introduction of the PEG chain increased the quantity of N atoms as clearly evidenced by the appearance of the peak of N 1s around 400 eV. High-resolution N 1s spectrum of GOTEG-N_3_ (Figure 1d, red curve, and Figure S3) shows three peaks. The peaks at 401.7 and 404.1 eV correspond to the negatively charged and positively charged nitrogen of the azido group, respectively.^35,36^ The third peak at 399.9 can be attributed to the amino function of the TEG chain. Following the click reaction, a remarkable increase of N1s peak centered at 399.8 eV, and corresponding to primary amines, secondary amines, and amide bonds of NPY peptide, was observed (Figure 1d, blue curve, Figure S3). The percentage of the nitrogen atoms on the surface of GO extracted from survey spectra were used to calculate the loading of the functional groups introduced at each step. The PEG chain and the NPY peptide were estimated at 400 μmol of azide functions (2.6% N 1s) and 130 μmol of peptide (12% N 1s) per g of material, respectively, corresponding to a yield of approximatively 33% in the azide transformation. This incomplete coupling may be due to the high steric hindrance of the NPY molecules onto the GO surface that then limits its drug loading.

The next step was to study the secondary structure of the peptide, in order to assess if the conjugation to GO maintains the original helical folding of the peptide.^37^ The conformation of NPY was investigated by CD (Figure 1e). The different molecules were dispersed in a mixture of TFE in PBS, typical condition that mimics the lipid bilayers and stabilizes the helical structure of peptides and proteins. NPY peptide shows the typical profile of the α-helix with the negative bands at 222 and 208 nm. Grafting NPY on the surface of GO does not affect the α-helical conformation of the natural peptide. The intensity of the two CD curves, allowed us to quantify the percentage of helical fraction (HF) of the peptide in the native and in the GO-grafted state. The value corresponding to NPY is 28%, in full agreement with the data reported in the literature.^38^ For GO–NPY, the calculated HF is 60%, more than doubling HF of native NPY. This increase might be attributed to a stabilization of the secondary structure of the peptide covalently linked to GO.

The enhancement of helicity after coupling a peptide to nanomaterials, exploiting either electrostatic interactions or click chemistry, has been demonstrated in previous studies^39−41^ and suggested a relationship between the surface of GO and the amino acids of the peptide, which interact to favor the formation of an α-helical conformation. Additionally, click chemistry was employed to rigidify a peptide to restrict its free conformation and to stabilize the α-helical structure on gold nanoparticles.^42^ In agreement with these previous observations, we detected an increase of HF of NPY upon coupling with the GO. However, even if the helicity might be involved in the receptor interaction, it is not the only portion of the peptide engaged in this recognition.^43,44^

**Figure 2:**
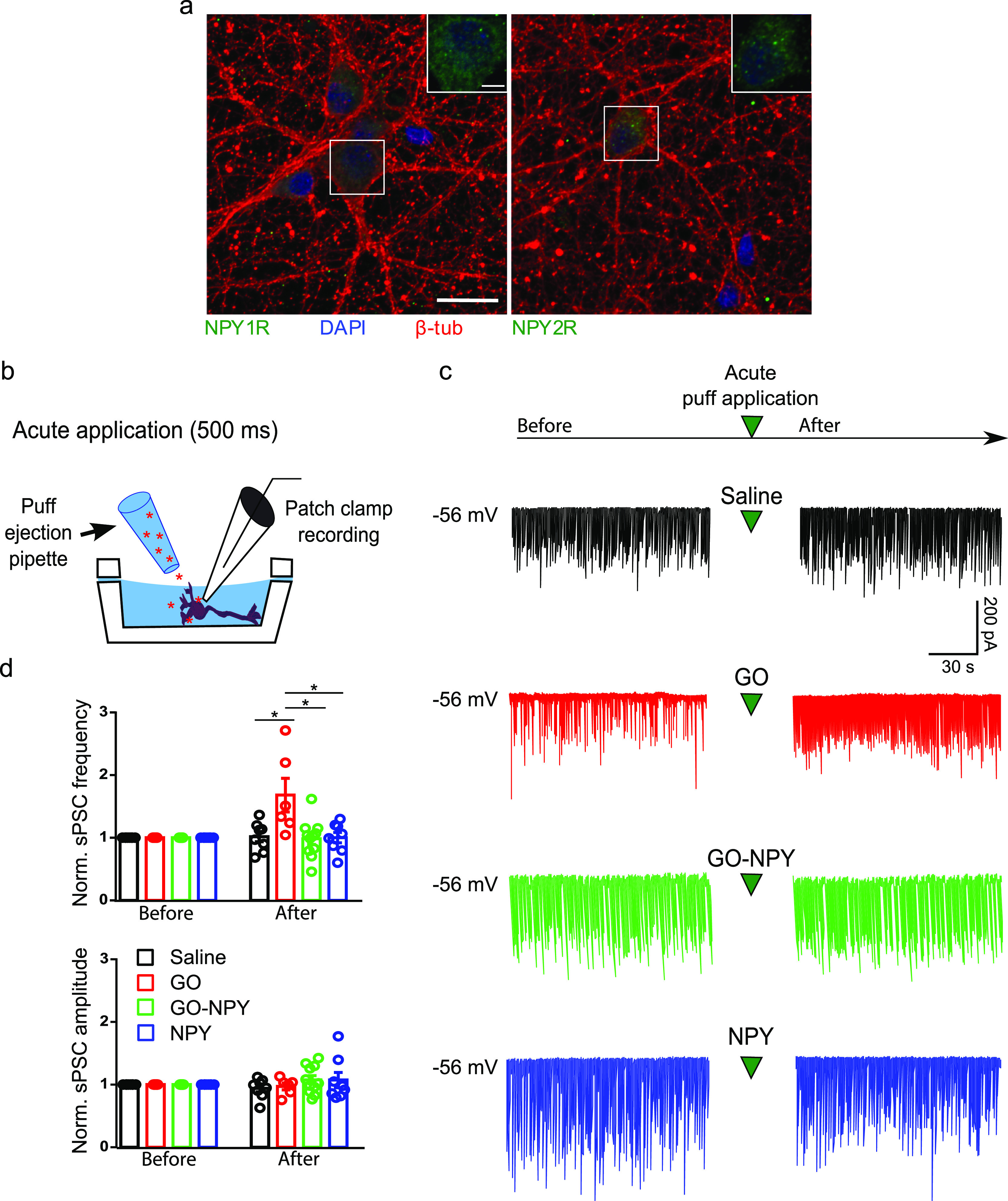
Effect of conjugate on neuronal activity of hippocampal cultures in acute applications. (a) Representative fluorescence microscopy images of hippocampal cultured cells showing the labeling for β-tubulin-positive neurons (in red) and for neuropeptide Y receptors (in green) type 1 (NPY1R; left) and type 2 (NPY2R; right), respectively. DAPI-positive nuclei (in blue). For each image, the area in the white squares was magnified in the upper right insets to better depict the somatic localization of receptors. Scale bar: 20 and 5 μm. (b) Experimental setting. (c) Exemplificative traces for different treatments (saline in black, GO in red, GO–NPY in green and NPY in blue) before (left column) and after (right column) their puff application. (d) Plots showing normalized sPSC frequency and amplitude for the different treatments. Note that only GO induce a post-application increase in sPSC frequency, *P < 0.05.

To understand the pharmacological responses of the prepared platform, we systematically compared the biological activity of the GO–NPY, GO, and pure NPY. We tested the impact of the NPY conjugates on dissociated hippocampal cultures. First, we evaluated the expression of NPY receptors in our model. As shown by immunofluorescence micrographs in Figure 2a, depicting hippocampal neurons co-labeled with antibodies against the specific neuronal marker β-tubulin III and against NPY receptor types 1 and 2, in this in vitro preparation both types of NPY receptors were expressed.

Next, we studied the effects of the conjugate on synaptic currents through the patch clamp technique. Because pristine GO flakes were reported to modify neuronal activity *per se*(15−19) the first set of experiments was aimed to understand if GO sheets, when conjugated to a peptide, retained their modulatory action on neurons. We exposed voltage-clamped neurons to short (500 ms) pressure ejections (puffs) of GO, GO–NPY, NPY, or saline solution, used as control, positioning a second pipette close to the recorded neuron (sketched in Figure 2b; see the Experimental Section(17,29)). This exposure is too brief to allow NPY modulation of the neuronal activity,^45−47^ while due to the nature of GO interactions with the synapses,^17^ in this type of application, GO induces a transient modulation of the synaptic event frequency.^17^ Dissociated hippocampal neurons display a prominent basal synaptic activity,^28^ documented by the occurrence of heterogeneous sPSCs characterized by variable amplitudes (Figure 2c). We applied puffs of saline solution alone or containing unconjugated GO (200 μg/mL), GO–NPY conjugates (200 μg/mL of nanomaterial corresponding to 10 μM of peptide), and NPY alone (10 μM, Figure 2c).

The traces reported in Figure 2c show that after the puff only unconjugated GO was able to tune the neuronal activity, while samples exposed to the other treatments did not present any change. We quantified the GO effect as an increment in the post-puff frequencies of sPSCs (normalized for the baseline pre-puff values) in neurons exposed to unconjugated GO respect to the other conditions (1.02 ± 0.08 control, *n* = 8, 1.7 ± 0.3 GO *n* = 6, 0.98 ± 0.09 GO–NPY, *n* = 11 and 1 ± 0.08 NPY, *n* = 8; see bar plot in Figure 2d). The differences were statistically significant (for control vs GO P = 0.0106, for GO vs GO–NPY *P* = 0.0039 and for GO vs NPY *P* = 0.0086). No variations were observed in the normalized post-puff sPSC amplitude upon all different treatments (0.94 ± 0.05 for control, 0.97 ± 0.05 for GO, 1.07 ± 0.07 for GO–NPY and 1.07 ± 0.12 for NPY, *P* > 0.05; Figure 2d). These experiments strongly suggest that covalently modified GO loses its ability to transiently regulate synaptic activity.^17^

**Figure 3:**
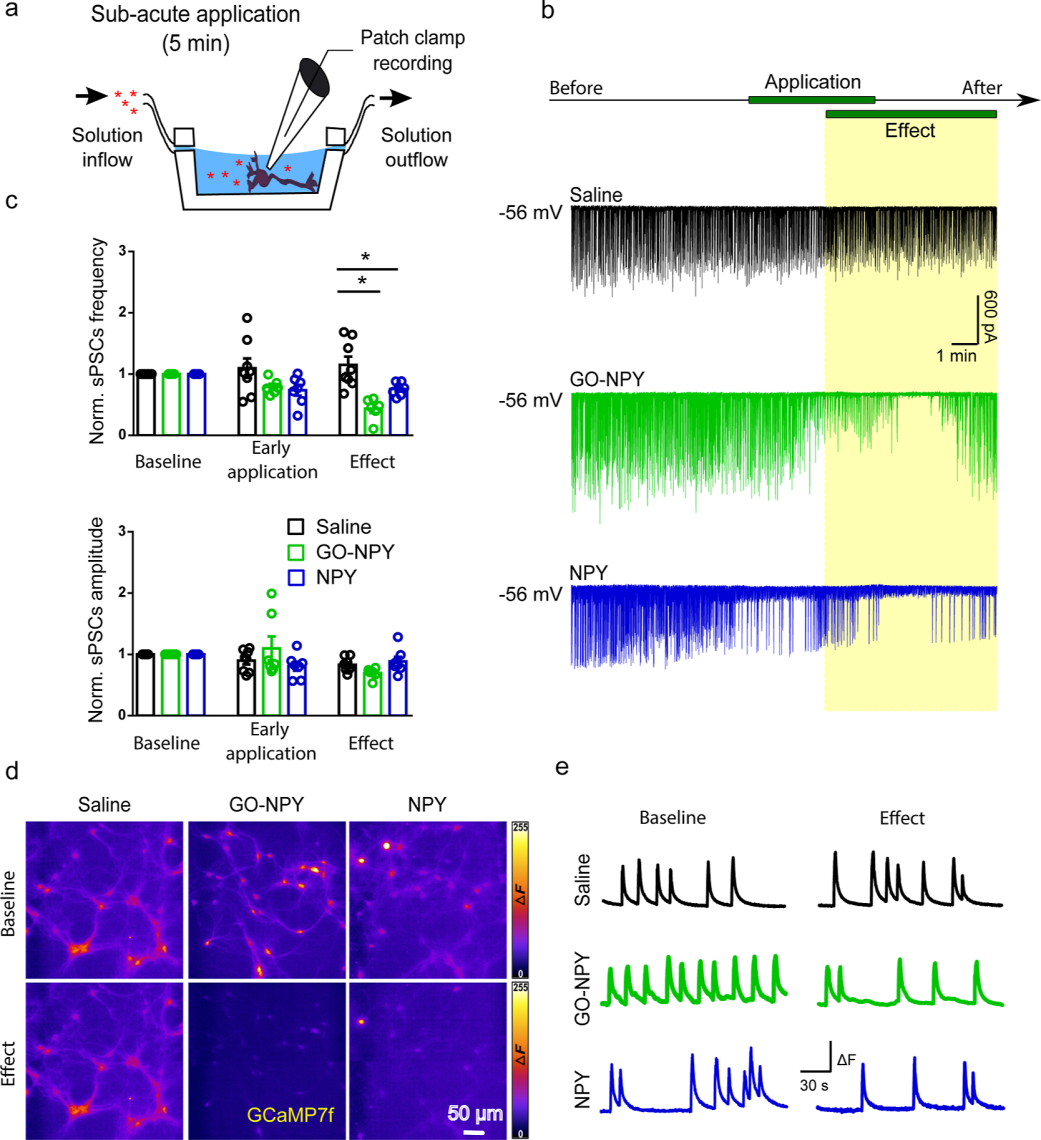
Effect of conjugate on neuronal activity of hippocampal cultures in sub-acute applications. (a) Experimental setting. (b) Exemplificative traces of recordings performed during different treatments (saline in black, GO–NPY in green, and NPY in blue) showing that NPY alone or in conjugation with GO induced a decrease in neuronal activity after some minutes from the beginning of peptide application. (c) Plots showing normalized sPSC frequency and amplitude for the different treatments during the first 3 min of application (early application) and when the effect of the peptide was consolidated (corresponding to the last 2 min of application together with the initial phase of wash out). Note that both GO–NPY and NPY induced a similar decrease in sPSC frequency. (d) GCaMP7f recordings with fluorescence levels in false colors during the baseline and effect periods (top and bottom panels, respectively) for the different conditions. (e) Fluorescence transient traces recorded for the different treatments (saline in black, GO–NPY in green, and NPY in blue) during the baseline and the effects periods. Note that NPY alone or in conjugation with GO induced a decrease in the occurrence of calcium transient after its administration (calcium transient frequencies were 0.017 and 0.019 Hz in control, 0.028 and 0.014 Hz in GO–NPY, and 0.022 and 0.011 Hz in NPY, baseline and effect, respectively). *P < 0.05.

In the second set of experiments, we investigated if NPY was still pharmacologically active after the binding to the nanomaterial. For this purpose, we used a longer time of application (5 min), compatible with the activation of NPY receptors,^45−47^ in which NPY (1 μM), alone or in conjugation with GO (20 μg/mL), was administered sub-acutely through the perfusion system while monitoring neuronal activity (Figure 3a). These recordings showed, after NPY or GO–NPY exposure, a decreased trend in the synaptic activity respect to controls (Figure 3b). Although the differences fall short for meeting statistical significance (*P* values > 0.05), such modulation could be detected already during the initial phase (3 min) of peptide/conjugate application as a progressive decrement of the normalized sPSC frequency when compared to control (1.09 ± 0.16 control *n* = 8, 0.79 ± 0.05 GO–NPY *n* = 6, and 0.73 ± 0.09 NPY, *n* = 7). Accordingly, PSC frequency decreased significantly during the last 2 min of the drug applications and remained low for the next 7 min of wash out (named effect phase and sketched in Figure 3b; 1.15 ± 0.13 control, 0.44 ± 0.07 GO–NPY and 0.76 ± 0.04 NPY). The differences were statistically significant for the control versus GO–NPY (*P* = 0.0002) and for the control versus NPY (*P* = 0.0245), while *P* was >0.05 for GO–NPY versus NPY (Figure 3b,c). No changes were observed in sPSC amplitudes (during initial application phase 0.90 ± 0.06 control, 1.10 ± 0.19 GO–NPY and 0.80 ± 0.07 NPY; during the “effect” phase 0.83 ± 0.04 for control, 0.69 ± 0.04 for GO–NPY and 0.86 ± 0.08 for NPY; Figure 3c, *P* values > 0.05). These data suggest that NPY bound to GO maintains its pharmacological activity and quantitatively depress neuronal activity as unconjugated NPY.

In order to evaluate the impact of the conjugates on multiple neurons simultaneously, we recorded live calcium responses in dissociated hippocampal cultures expressing a genetically encoded Ca^2+^ indicator based on green fluorescent protein fluorescence (GCaMP7f). In the presence of gabazine (10 μM), a specific antagonist of GABA_A_ receptors, to synchronize network activity and enhance coordinated calcium transients, we applied the different treatments sub-acutely. As shown in Figure 3d,e, before the treatments (baseline) in all the conditions hippocampal neurons exhibited similar repetitive GCaMP7f fluorescent transients, indicative for a synchronized neuronal network activity. When NPY was applied sub-acutely, alone or in conjugation with GO, it induced a reduction in the occurrence of calcium transients, while in the control condition (saline), no changes were observed. Through this experiment, we confirmed that NPY was biologically active in depressing neuronal activity at a network level after binding to the nanomaterial.

**Figure 4:**
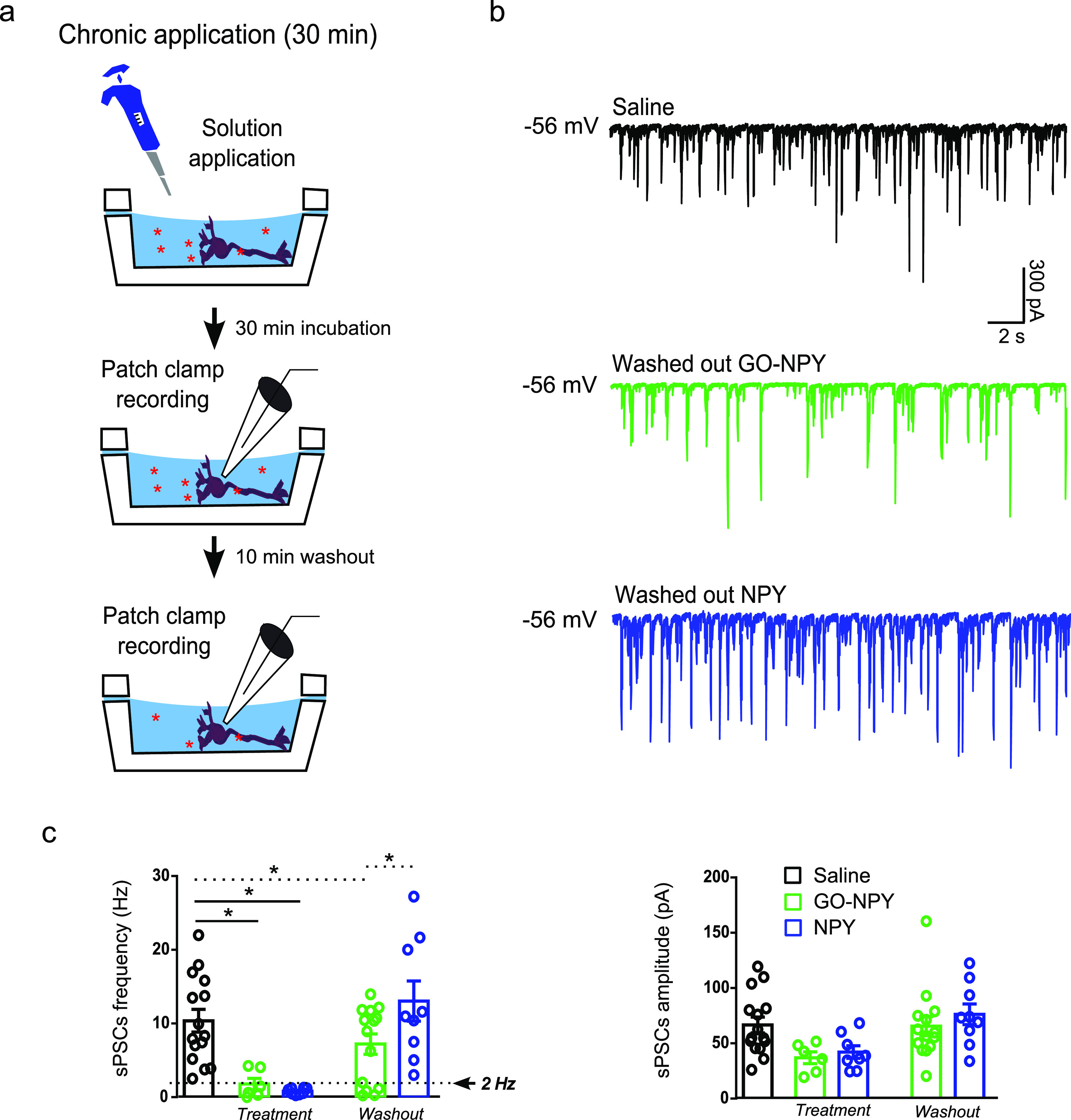
Effect of conjugate on neuronal activity of hippocampal cultures in cronic applications and wash out. (a) Experimental setting. (b) Exemplificative traces of recordings performed after wash out of different treatments (saline in black, GO–NPY in green and NPY in blue) showing that NPY conjugated with GO presented a residual inhibitory action on neuronal activity respect to free NPY. (c) Plots showing sPSC frequency and amplitude for the different treatments during the incubation and after wash out. Note that both GO–NPY and NPY induced a similar decrease in sPSC frequency during the incubation; however, only in cultures exposed to GO–NPY, a subset of neurons presented sPSC frequency lower than 2 Hz after wash out, *P < 0.05.

Finally, we characterized the effect of GO–NPY in chronic (30 min) incubation. In this set of experiments, cultured cells were exposed to GO–NPY (20 μg/mL and 1 μM), NPY (1 μM), or saline, prior to electrophysiological recordings (Figure 4a). Confirming results obtained in the sub-acute application experiments, we observed a comparable reduction in sPSC frequency in cultures treated with NPY alone (0.8 ± 0.1 Hz, *n* = 8) or with GO–NPY (1.8 ± 0.8 Hz, *n* = 6) respect to controls (10.4 ± 1.5 Hz, *n* = 15; Figure 4b). The differences were statistically significant for control versus GO–NPY (*P* = 0.0338) and for control versus NPY (*P* = 0.0022), but not between GO–NPY and NPY (*P* > 0.05). No changes in sPSC amplitudes were found among the different treatments (66 ± 7 pA control, 37 ± 5 pA GO–NPY and 42 ± 6 pA NPY, Figure 4c).

In another set of experiments, the 30 min drug applications were followed by 10 min washout to further investigate the duration of NPY effects when conjugated or when alone. Figure 4c shows that upon washout, 36% of cells (*n* = 14) treated with GO–NPY still displays depressed (<2 Hz) sPSC frequency, while in NPY-treated ones (*n* = 9), we never detected residual sPSC inhibition and frequency values were comparable to controls (*n* = 15). The differences between the groups were statistically significant (with two tailed Chi square test for control vs GO–NPY *P* = 0.011 and for GO–NPY vs NPY *P* = 0.0427; Figure 4c).

These experiments indicated that GO–NPY maintained its biological activity in chronic incubation, with a prolonged effect upon washout. This might indicate that in the conjugate, GO modifies the pharmacokinetic properties of NPY, favoring its prolonged effect in the place of action.

## Discussion

Because GO has been recently reported to tune neuronal activity,^15−19^ the aim of our study was to investigate whether when this nanomaterial is engineered in a drug delivery context it could contribute to the biological activity of the conjugates. The starting point was to determine the type of linkage between the GO and NPY peptide. For this purpose, we exploited the covalent functionalization of the oxygen moieties present on the surface of GO. The advantage of this type of bond is to avoid undesired release of the molecules, likely occurring when they are physiosorbed. Additionally, physisorption can also alter the structure of the biomolecules,^48^ eventually decreasing its biological activity. The latter effect is crucial for complex biomolecules such as peptides where their biological activity is strictly dependent on their conformation in solution. Finally, the synthetic strategy should be adapted to the nanomaterial used and the molecule of interest chosen. In this case, to avoid reduction of the surface of GO (with an unavoidable decrease of the colloidal stability in aqueous media), it was necessary to use mild conditions, with reactions conducted at RT. GO was therefore covalently conjugated to NPY peptide via epoxide ring opening followed by a selective click reaction. By this strategy, we achieved the preparation of GO–NPY adduct with high drug loading. Comparing previous results, GOTEG-N_3_ epoxide opening reaction was higher than the one we reported with other small amines.^49^ Few works have described the functionalization of GO with peptides through click chemistry; however, without reporting the reaction yields.^50,51^ In this study, we have explored a facile method for covalent functionalization of the GO with high yield that does not alter the NPY conformation.

Next, we have characterized the biological activity of the GO–NPY in dissociated brain cell cultures. Our experiments showed that GO can be used as a bio-tolerable nano-carrier for drug delivery because once covalently conjugated with a neuro active compound, the conjugate is still driven to target neuronal membranes, where NPY receptors are expressed. In addition, GO preserves and prolongs NPY biological activity, possibly also through a stabilization of its secondary structure,^42^ while the nanomaterial effects, as synaptic modulator, are neutralized. This understanding was achieved by investigating the impact of the conjugates administered through applications of different durations, namely, acute, sub-acute, and chronic, which allowed dissecting the biological effects exerted by the nanomaterial from those of the peptide. To avoid differences in biological effects arising from diverse NPY doses, the used concentration of the peptide, alone or in conjugation with GO, was selected to be oversaturating for the modulation of synaptic activity.^52^

GO was previously reported to modulate neuronal activity with a biphasic behavior.^15,17,19,29^ In detail, GO locally applied to neurons through a brief puff of solution induce a transient increment in the frequency of sPSCs, caused by GO flakes interfering with the release of neurotransmitter containing pre-synaptic vesicles.^17,29^ In our experiments, we replicated such an effect for GO, while for GO–NPY, no changes were observed. Because NPY requires at least some minutes of application to induce a detectable biological response,^45−47^ it is reasonable to hypothesize that the lack of synaptic effects of the conjugated GO was not caused by the pharmacological activity of NPY, damping the nanomaterial-induced increment of neuronal activity. Because conjugation inevitably changes the material surface chemistry groups (Scheme 1 and Figure 1c), this may cause the loss of biological activity of the GO flakes *per se*.^17^ Based on these results, we suggest that GO when chemically modified is not anymore able to interfere with synaptic transmission and it can be considered as an inert platform for drug delivery.

In designing the conjugate, we selected NPY as the molecule to bind GO for several reasons. In particular, this peptide has been reported to exert a modulatory action on synaptic transmission.^21,23,53,54^ In our work, this feature was exploited as a readout to verify if NPY retained its pharmacological activity once conjugated to GO. From this perspective, as a model for our experiments, we used dissociated hippocampal cultures expressing NPY receptors,^55−57^ as confirmed by the immunostaining. Through electrophysiological recordings we monitored synaptic activity, which was modulated similarly by conjugated and unconjugated NPY, strongly suggestive for the preservation of NPY biological activity once bound to the nanoplatform. Regarding the mechanisms of action of NPY, alone or in conjugation with the nanomaterials, we observed that both sub-acute and chronic exposures induced the expected downregulation of synaptic transmission. This was consistent with previously reported literature,^46,47,56,58^ and suggests that NPY effects are mainly mediated by the Y2 receptors presynaptic inhibition of neurotransmitters release.^47,58,59^ In live calcium imaging experiments (Figure S4), the sub-acute application of NPY in the presence of BIIE0246, a specific antagonist of Y2 receptors,^52^ exerted virtually no effect on neuronal signaling, confirming that in our cultures NPY acted mainly via activation of this receptor type.

NPY-induced modulation of neuronal activity presented a slower kinetic of onset respect to that of GO, allowing to isolate the effects due to the nanomaterial, as discussed above. In addition, in the last years, NPY has emerged as a potential therapeutic molecule for the treatment of several neuropathologies, including neurodegenerative disorders,^60^ epilepsy,^61,62^ and anxiety diseases.^63,64^ The use of graphene-based drug delivery strategies might favor its translation in medicine, overcoming some current issues related to its pharmacokinetics, such as the short half-life.^65^ In agreement with this scenario, our washout experiments after chronic incubation revealed a residual effect of NPY conjugated to the nanomaterial respect to the unconjugated peptide, suggesting that the conjugation with GO could promote the permanence of NPY in proximity of the synapses, thus augmenting the duration of the therapeutic effect of the peptide. Further investigations will be necessary to assess how GO–NPY could modulate synaptic activity in vivo to support the future use of GO for the development of smart platforms for nervous system drug delivery.

## Conclusions

In summary, our experiments characterized GO as a nanocarrier preserving and prolonging the pharmacological activity of the bound molecule. From this perspective, the loss of GO modulatory effects *per se* on neuronal transmission, probably due to the covalent modification of the nanomaterial, makes GO an inert platform for drug delivery. However, other strategies of complexation between GO and the active compound might be explored in the future for developing multifunctional nanotools enabling both the nanomaterial and the drug to exert a combined therapeutic action.

## Supporting Information Available

Relative XPS atomic percentage of C, O, and N; FTIR spectra of GO and GOTEG-N_3_; SEM images of nanomaterials; detailed high-resolution scans of N 1s of GO, GOTEG-N_3_, and GO–NPY; and live calcium imaging experiments (PDF)

## Notes

The data used to support the findings of this study are available from the corresponding author upon request.
