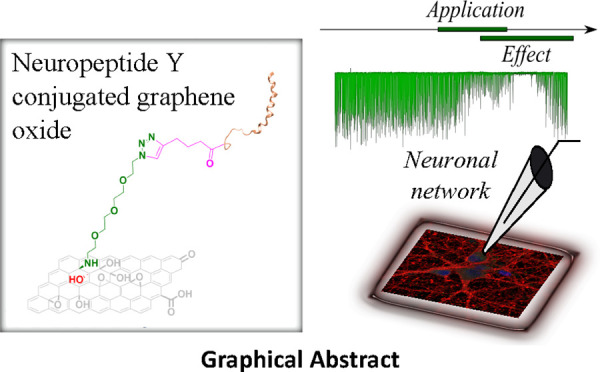# Correction to “Bonding of Neuropeptide Y on Graphene Oxide for Drug Delivery Applications to the Central Nervous System”

**DOI:** 10.1021/acsanm.3c01600

**Published:** 2023-05-02

**Authors:** Giada Cellot, Lucas Jacquemin, Giacomo Reina, Audrey Franceschi Biagioni, Mario Fontanini, Olivier Chaloin, Yuta Nishina, Alberto Bianco, Laura Ballerini

In the published paper, there is a mistake in the structure of the final GO–NPY conjugate both in Scheme 1 and in the graphical abstract. A corrected version of the scheme and the graphical abstract appear below.

**Scheme 1:**
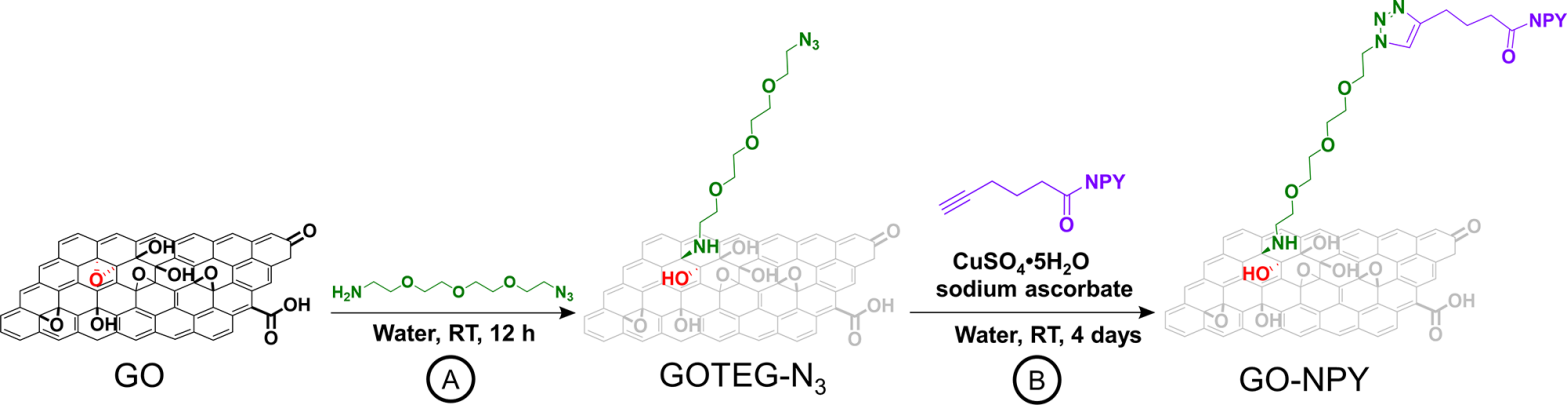
Functionalization of GO by Epoxide Ring Opening (A), Followed by the Copper Catalyzed Click Reaction (B)