# Supplementary material for: Bonding of Neuropeptide Y on Graphene Oxide for Drug Delivery Applications to the Central Nervous System
Source: ACS Appl Nano Mater. 2022 Dec 2;5(12):17640–51. doi: 10.1021/acsanm.2c03409 (PMC9791619; doi:10.1021/acsanm.2c03409)
Supplement: Supplementary file 1 — an2c03409_si_001.pdf [file an2c03409_si_001.pdf]

Supplementary Information

Bonding of Neuropeptide Y on Graphene Oxide for Drug Delivery Applications to the Central Nervous System

Giada Cellot[1*], Lucas Jacquemin[2], Giacomo Reina[2$], Audrey Franceschi Biagioni[1], Mario Fontanini[1], Olivier Chaloin[2], Yuta Nishina[3], Alberto Bianco[2*], Laura Ballerini[1*]

[1]International School for Advanced Studies, SISSA, Via Bonomea n.265, 34136, Trieste, Italy

[2]CNRS, Immunology, Immunopathology and Therapeutic Chemistry, UPR 3572, University of Strasbourg ISIS, 67000 Strasbourg, France.

[3]Graduate School of Natural Science and Technology and Research Core for Interdisciplinary Sciences, Okayama University, Tsushimanaka, Kita-ku, Okayama 700-8530, Japan.

*Corresponding authors: cellot@sissa.it, laura.ballerini@sissa.it, a.bianco@ibmc-cnrs.unistra.fr

[$]Current address: Swiss Federal Laboratories for Materials Science and Technology, Lerchenfeldstrasse 5, 9014 St. Gallen, Switzerland.

Table S1. Table reporting the relative XPS atomic percentage of C, O and N.

Element	GO [%]	GOTEG-N_3 [%]	GO-NPY [%]
C	69.1±0.1	66.5±0.1	65.4±0.3
O	30.4±0.2	30.4±0.1	21.3±0.1
N	0.5±0.2	3.0±0.1	12.0±0.2

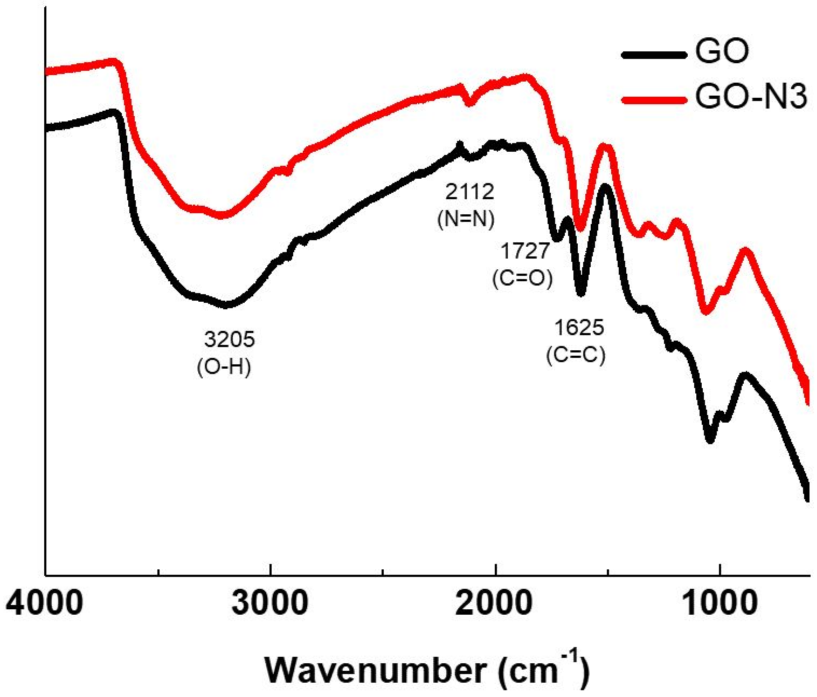

Figure S1. FTIR spectra of GO and GOTEG-N_3.

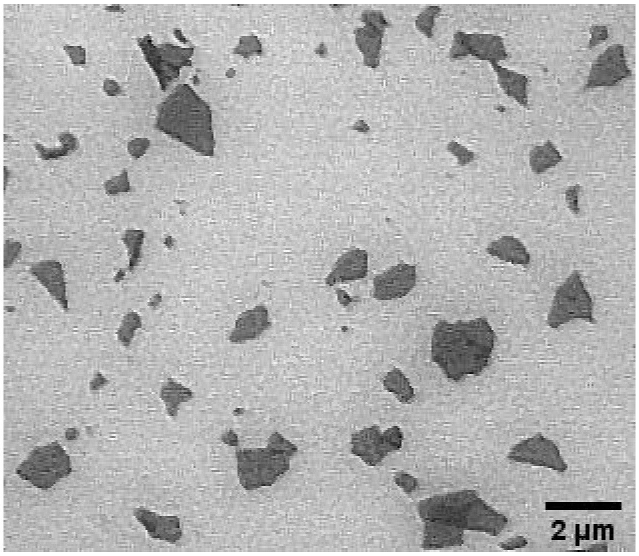

Figure S2. SEM image of GO (×5.00K).

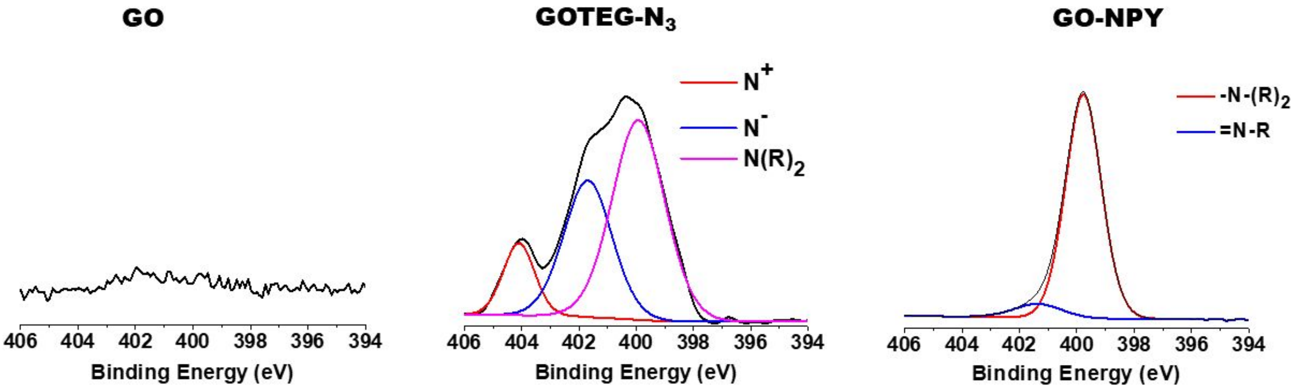

Figure S3. The detailed high-resolution scans of N1s of GO, GOTEG-N_3, and GO-NPY.

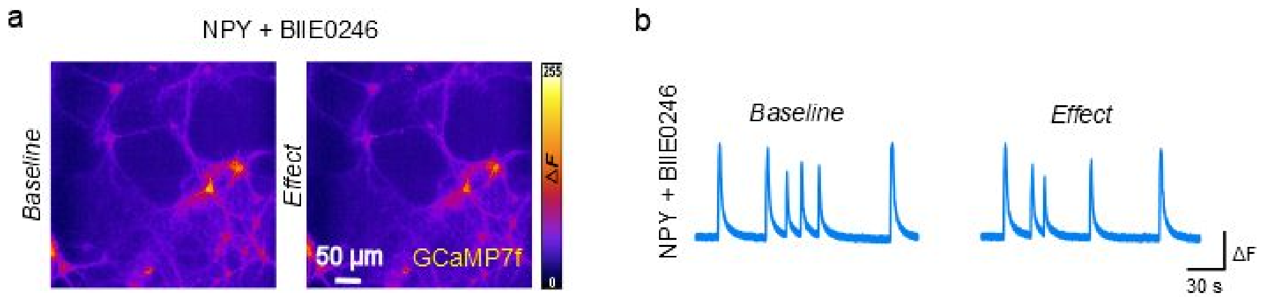

Figure S4. Live calcium imaging experiments. (a) GCaMP7f recordings with fluorescence levels in false colors during the baseline and effect periods (left and right panels, respectively) for NPY treatment in the presence of the Y2 receptor antagonist BIIE0246. (b) Fluorescence transient traces recorded for NPY treatment in the presence of Y2 receptor antagonist BIIE0246. Note the lack of changes in the occurrence of calcium transients during the effect phase respect to the baseline, indicating that NPY acted mainly through Y2 receptors (calcium transient frequencies were 0.017 Hz and 0.014 Hz, baseline and effect respectively).
